# The Potential of Semaglutide Once-Weekly in Patients Without Type 2 Diabetes with Weight Regain or Insufficient Weight Loss After Bariatric Surgery—a Retrospective Analysis

**DOI:** 10.1007/s11695-022-06211-9

**Published:** 2022-07-25

**Authors:** Anne Lautenbach, Marie Wernecke, Tobias B. Huber, Fabian Stoll, Jonas Wagner, Sebastian M. Meyhöfer, Svenja Meyhöfer, Jens Aberle

**Affiliations:** 1 III Department of Medicine, University Medical Center Hamburg-Eppendorf, Martinistr. 52, 20246 Hamburg, Germany; 2 Department of General, Visceral and Thoracic Surgery, University Medical Center Hamburg-Eppendorf, 20246 Hamburg, Germany; 3 Institute for Endocrinology & Diabetes, University of Lübeck, 23562 Lübeck, Germany; 4 German Center for Diabetes Research (DZD), 85764 Neuherberg, Germany; 5 First Department of Medicine Endocrinology and Diabetes, University Clinic Schleswig-Holstein – Campus Lübeck, 23538 Lübeck, Germany

**Keywords:** Semaglutide, GLP-1 receptor agonist, Weight regain, Bariatric surgery

## Abstract

**Purpose:**

About 20–25% of patients experience weight regain (WR) or insufficient weight loss (IWL) after bariatric metabolic surgery (BS). Therefore, we aimed to retrospectively assess the effectiveness of adjunct treatment with the GLP-1 receptor agonist semaglutide in non-diabetic patients with WR or IWL after BS.

**Materials and Methods:**

Post-bariatric patients without type 2 diabetes (T2D) with WR or IWL (*n* = 44) were included in the analysis. The primary endpoint was weight loss 3 and 6 months after initiation of adjunct treatment. Secondary endpoints included change in BMI, HbA1c, lipid profile, hs-CRP, and liver enzymes.

**Results:**

Patients started semaglutide 64.7 ± 47.6 months (mean ± SD) after BS. At initiation of semaglutide, WR after post-bariatric weight nadir was 12.3 ± 14.4% (mean ± SD). Total weight loss during semaglutide treatment was − 6.0 ± 4.3% (mean ± SD, *p* < 0.001) after 3 months (3.2 months, IQR 3.0–3.5, *n* = 38) and − 10.3 ± 5.5% (mean ± SD, *p* < 0.001) after 6 months (5.8 months, IQR 5.8–6.4, *n* = 20). At 3 months, categorical weight loss was > 5% in 61% of patients, > 10% in 16% of patients, and > 15% in 2% of patients. Triglycerides (OR = 0.99; *p* < 0.05), ALT (OR = 0.87; *p* = 0.05), and AST (OR = 0.89; *p* < 0.05) at baseline were negatively associated with weight loss of at least 5% at 3 months’ follow-up (*p* < 0.05).

**Conclusion:**

Treatment options to manage post-bariatric excess weight (regain) are scarce. Our results imply a clear benefit of adjunct treatment with semaglutide in post-bariatric patients. However, these results need to be confirmed in a prospective randomized controlled trial to close the gap between lifestyle intervention and revision surgery in patients with IWL or WR after BS.

**Graphical abstract:**

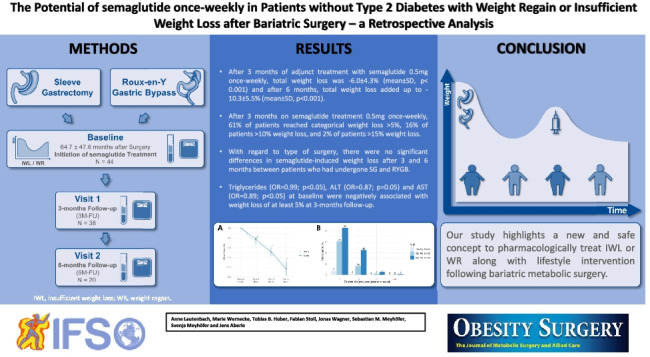

## Introduction/Purpose

There is an urgent need to develop novel strategies to treat insufficient weight loss or weight regain in post-bariatric patients. Despite the overall safety and efficacy of bariatric metabolic surgery (BS), outcomes vary considerably for individual patients [1]. Approximately 20–25% of patients experience considerable weight regain (WR) defined as regain of weight that occurs after achievement of an initial successful weight loss (defined as EWL% > 50%) or insufficient weight loss (IWL) defined as < 50% EWL at 18 months after BS [2, 3]. As a consequence, patients may only experience partial remission of comorbidities; e.g., a large number of patients who experience complete T2D remission in the early period after surgery suffer a relapse on long-term follow-up [4]. In the Swedish Obese Subjects (SOS) study, the first long-term prospective trial to provide information on the effects of BS, 10% of RYGB patients did not maintain ≥ 5% weight loss at 10-year follow-up [1, 5, 6]. The rate of conversion surgery due to failure of the initial procedure is almost twice after sleeve gastrectomy (SG) than after Roux-en-Y bypass (RYGB) [7]. Conversion surgery usually implies a greater degree of malabsorption and higher rate of morbidity, reoperations, and readmission [8].

The Endocrine Society’s clinical practice guidelines on the management of the post-bariatric surgery patient recommend that pharmacotherapy should be included in the multidisciplinary treatment of WR [1, 9]. However, most studies evaluating pharmacotherapy for long-term treatment of overweight and obesity have excluded participants with a history of BS [10, 11]. Thus, there are no approved weight management drugs for use in patients who had BS, even though several studies have suggested that the combination of pharmacotherapy and lifestyle modification results in more weight loss than either approach used alone [1, 12, 13]. So far, there is limited data about the efficacy of pharmacotherapy to treat insufficient weight loss or weight regain in the post-bariatric population. Overall, the currently available FDA-approved weight loss drugs have been associated with relatively modest weight loss in post-bariatric patients [14, 15].

In 2021, the long-acting human GLP-1 receptor agonist (GLP-1 RA) semaglutide was approved for the treatment of obesity in the USA and in 2022 in Europe. In non-surgical patients with obesity, semaglutide induces clinically important weight loss and improvement in cardiometabolic risk factors [16]. So far, there are no data on the effectiveness of semaglutide in the management of IWL and WR in non-diabetic patients after BS.

The aim of the present study was to assess the potential effects of semaglutide 0.5 mg once-weekly in non-diabetic patients with WR and IWL as adjunct therapy after BS in a retrospective setting.

## Materials and Methods

### Study Population

A total of 53 adult patients (≥ 18 years) who underwent either sleeve gastrectomy (SG) or Roux-en-Y gastric bypass (RYGB) according to the S3 Leitlinie (Guidelines) *Chirurgie der Adipositas* [17] were included in this study. Patients with second step procedures prior to adjunct treatment with semaglutide were considered having SG (*n* = 1). Between 2020 and 2022, patients attended our obesity outpatient clinic, which is certified by the European Accreditation Council for Bariatric Surgery as a center of excellence for obesity and metabolic surgery.

Patients with WR defined as continuous WR after an initial successful weight loss (defined as EWL > 50%) and IWL defined as achieving a nadir weight with EWL < 50% after surgery were prescribed semaglutide in addition to autonomous lifestyle modification for weight management following BS [2, 3]. All patients gave their informed consent to off-label drug use prior to prescription. Semaglutide was administered with a prefilled pen injector at a dose of 0.25 mg/week for the first 4 weeks and increased to 0.5 mg/week thereafter according to individual tolerability. Contraindications for use were a history of acute/chronic pancreatitis and pancreatic cancer, positive family history of multiple endocrine neoplasia, current pregnancy/breastfeeding, and glomerular filtration rate below 15 mL/min. Exclusion criteria included incomplete records, presence of T2D, and revision surgery during follow-up. Of an initial population of 53 patients, 9 patients were excluded from the final analysis (*n* = 44): Two patients were excluded due to coexisting T2D, and two patients discontinued treatment with semaglutide due to revision surgery or pregnancy occurring during follow-up. In 3 patients, treatment was discontinued 4 weeks after treatment initiation due to lack of effectiveness (defined as total weight loss < 2 kg within 4 weeks), and 2 patients were lost to follow-up.

### Study Design

Follow-up data were retrospectively collected from 44 patients at baseline. To provide reasonable comparability between the cases, the available data were allocated 3 “visits” by time in relation to initiation of semaglutide once-weekly. In addition to baseline data 64.7 ± 47.6 months (mean ± SD) after surgery, data from visit 1 (*n* = 44) were analyzed 1 month after treatment initiation, data from visit 2 (*n* = 38) 3 months after treatment initiation, and data from visit 3 (*n* = 20) 6 months after treatment initiation with semaglutide.

### Variables

Data on height (cm), weight (kg), body mass index (BMI; kg/m^2^), sex, age, type of surgery, platelets (10^3^/µL), CRP (mg/L), hs-CRP (mg/dL), hemoglobin (g/dL), albumin (g/L), aspartate aminotransferase (AST; U/L), alanine aminotransferase (ALT; U/L), gamma-glutamyl transpeptidase (GGT; U/L), total cholesterol (TC; mg/dL), triglycerides (mg/dL), high-density lipoprotein cholesterol (HDL; mg/dL), low-density lipoprotein cholesterol (LDL; mg/dL), lipase (U/L), and HbA1c (%) were analyzed at baseline and during follow-up. Percent of total weight loss (%TWL) was calculated by dividing the difference between initial weight and postoperative weight by initial weight multiplied by 100 [18]. Clinically significant weight loss following medical treatment adjunct to BS was defined as > 5% decrease in body weight as per clinical guidelines [19, 20]. Side effects were pulled from electronic medical records.

### Statistical Methods

Standard descriptive statistics were used for all study end points. Distributions of continuous variables were described with mean and standard deviation (SD). Categorical variables were described with absolute and relative frequencies. Continuous variables at baseline were compared using Student’s *t*-test. Categorical variables were compared using chi-square statistics. Continues data between multiple visits were compared using one-way ANOVA with Dunnett’s test for multiple comparisons. Weight loss outcomes between subgroups and multiple visits were compared using two-way ANOVA with Šídák’s test for multiple comparisons. Univariate logistic regression analysis was conducted to identify significant factors of successful weight loss defined as relative weight loss of ≥ 5% at 3 months’ follow-up compared to the baseline visit. Odds ratio (OR) with 95% confidence intervals (CI) and *p* values were calculated. *p* values below 0.05 were considered statistically significant.

## Results

### Patient Characteristics at Baseline

Baseline characteristics are presented in Table 1. Age was 46.4 ± 8.8 years (mean ± SD), and 75.4% of patients were female. 65.9% of patients underwent SG, and 34.1% underwent RYGB as initial weight loss procedure. BMI prior to surgery was 49.4 ± 8.9 kg/m^2^ (mean ± SD), total weight loss after surgery was –21.5 ± 10.3% (mean ± SD), and maximum weight loss following BS was − 29.1 ± 11.9% (mean ± SD). Postoperative WR after post-bariatric weight nadir and before adjunct semaglutide was 12.3 ± 14.4% (mean ± SD).

**Table 1:** Anthropometric and biochemical characteristics at baseline by type of surgery

	**RYGB (*N* = 15)**	**SG (*N* = 29)**	**Total (*N* = 44)**	***p* value**
Age [year]	46.8 (7.3)	46.3 (9.6)	46.4 (8.8)	0.859
Sex (females)	12 (80%)	20 (69%)	32 (73%)	0.436
Body weight before BS [kg]	136.3 (17.5)	150.5 (38.7)	145.7 (33.5)	0.185
BMI before BS [kg/m^2^]	48.0 (5.9)	50.1 (10.1)	49.4 (8.9)	0.467
Body weight nadir post BS [kg]	92.5 (18.9)	107.9 (27.2)	102.9 (25.6)	0.065
BMI nadir post BS [kg/m^2^]	32.5 (6.0)	35.8 (6.5)	34.7 (6.5)	0.118
Time from BS to weight loss nadir [months]	27.8 (20.1)	28.2 (45.9)	28.0 (39.1)	0.973
Time from BS to initiation of semaglutide treatment [months]	78.8 (37.8)	57.4 (51.1)	64.7 (47.6)	0.160
Time from weight nadir to initiation of semaglutide treatment [months]	50.8 (32.5)	29.2 (32.7)	36.2 (33.8)	0.048
Total weight loss from BS to nadir [%]	− 32.7 (10.3)	− 27.4 (12.4)	− 29.1 (11.9)	0.172
Weight regain from nadir to initiation of semaglutide treatment [%]	17.4 (15.8)	9.8 (13.2)	12.3 (14.4)	0.103
Weight prior to initiation of semaglutide treatment [kg]	106.5 (18.2)	117.1 (27.7)	113.5 (25.2)	0.187
BMI prior to initiation of semaglutide treatment [kg/m^2^]	37.3 (6.4)	38.9 (6.5)	38.3 (6.4)	0.465
Hb prior to initiation of semaglutide treatment [g/dL]	12.9 (1.2)	13.7 (1.3)	13.4 (1.3)	0.066
Platelets prior to initiation of semaglutide treatment [Mrd/L]	288.8 (93.7)	256.7 (55.2)	267.7 (71.3)	0.160
HbA1c prior to initiation of semaglutide treatment [%]	5.4 (0.4)	5.3 (0.3)	5.3 (0.4)	0.424
Albumin prior to initiation of semaglutide treatment [g/L]	39.3 (2.2)	38.5 (3.6)	38.8 (3.2)	0.402
Total cholesterol prior to initiation of semaglutide treatment [mg/dL]	176.1 (28.8)	185.8 (38.8)	182.4 (35.6)	0.398
Triglycerides prior to initiation of semaglutide treatment [mg/dL]	149.9 (81.5)	151.6 (62.8)	151.0 (68.8)	0.940
HDL-cholesterol prior to initiation of semaglutide treatment [mg/dL]	61.3 (20.9)	52.3 (10.1)	55.3 (15.1)	0.060
LDL-cholesterol prior to initiation of semaglutide treatment [mg/dL]	84.9 (17.2)	100.8 (35.1)	95.4 (30.9)	0.107
Total cholesterol/HDL-cholesterol ratio prior to initiation of semaglutide treatment	3.0 (1.0)	3.7 (0.9)	3.4 (1.0)	0.047
AST prior to initiation of semaglutide treatment [U/L]	25.1 (6.7)	19.9 (5.8)	21.7 (6.5)	0.011
ALT prior to initiation of semaglutide treatment [U/L]	27.2 (11.4)	21.2 (8.8)	23.2 (10.1)	0.060
GGT prior to initiation of semaglutide treatment [U/L]	17.1 (10.2)	22.4 (16.7)	20.6 (14.9)	0.260
CRP prior to initiation of semaglutide treatment [mg/L]	2.2 (2.1)	5.0 (6.2)	4.1 (5.4)	0.134
hs-CRP prior to initiation of semaglutide treatment [mg/dL]	0.2 (0.2)	0.4 (0.5)	0.3 (0.4)	0.134
Lipase prior to initiation of semaglutide treatment [U/L]	42.7 (11.9)	42.5 (14.2)	42.5 (13.3)	0.966
Total weight loss following BS to initiation of semaglutide treatment [%]	22.0 (8.2)	21.2 (11.4)	21.5 (10.3)	0.803
Prediabetes prior to initiation of semaglutide treatment	5 (33%)	3 (10%)	8 (18%)	0.061

Data are reported as mean (SD) and *N* (%). *N*, number of individuals; *SG*, sleeve gastrectomy; *RYGB*, Roux-en-Y gastric bypass; *BS*, bariatric metabolic surgery; *Hb*, hemoglobin; *HDL*, high-density lipoprotein; *LDL*, low-density lipoprotein; *AST*, aspartate aminotransferase; *ALT*, alanine aminotransferase; *GGT*, gamma-glutamyl transpeptidase; *CRP*, C-reactive protein; *hs-CRP*, high sensitive C-reactive protein

### Weight Loss Outcomes

Patients initiated semaglutide 64.7 ± 47.6 months (mean ± SD) after bariatric metabolic surgery at a BMI of 38.3 ± 6.4 kg (mean ± SD). After 3 months (3.2, IQR 3.0–3.5, *n* = 38) of treatment with semaglutide, total weight loss was − 6.0 ± 4.3% (mean ± SD, *p* < 0.001) and after 6 months (5.8, IQR 5.8–6.4, *n* = 20), total weight loss added up to − 10.3 ± 5.5% (mean ± SD, *p* < 0.001).

After 3 months on semaglutide treatment, 61% of patients reached categorical weight loss > 5%, 16% of patients > 10% weight loss, and 2% of patients > 15% weight loss. After 6 months of adjunct semaglutide treatment, 85% of patients reached > 5% weight loss, 45% of patients reached > 10% weight loss, and 5% of patients reached > 15% weight loss.

Female patients presented a more pronounced weight loss at 3 months’ (− 7.10 ± 3.95%) and 6 months’ follow-up (− 11.04 ± 5.74%) after initiation of adjunct semaglutide treatment once-weekly compared to male patients (− 2.44 ± 3.77% and − 5.90 ± 2.87%, respectively). This difference in weight loss outcomes was statistically significant for the 3-month follow-up visit (*p* = 0.005) (data not shown).

68.2% of patients were classified as having WR, and 31.8% of patients were classified as having IWL. Differentiating between these two groups, no significant differences in weight loss response were found (data not shown).

Side effects included nausea during the first 2 weeks of treatment in two patients. In 1 patient, increase in pancreatic lipase levels was observed, which resolved spontaneously 6 months after treatment initiation.

### Weight Loss Outcomes Depending on Type of Surgery

**Fig. 1:**
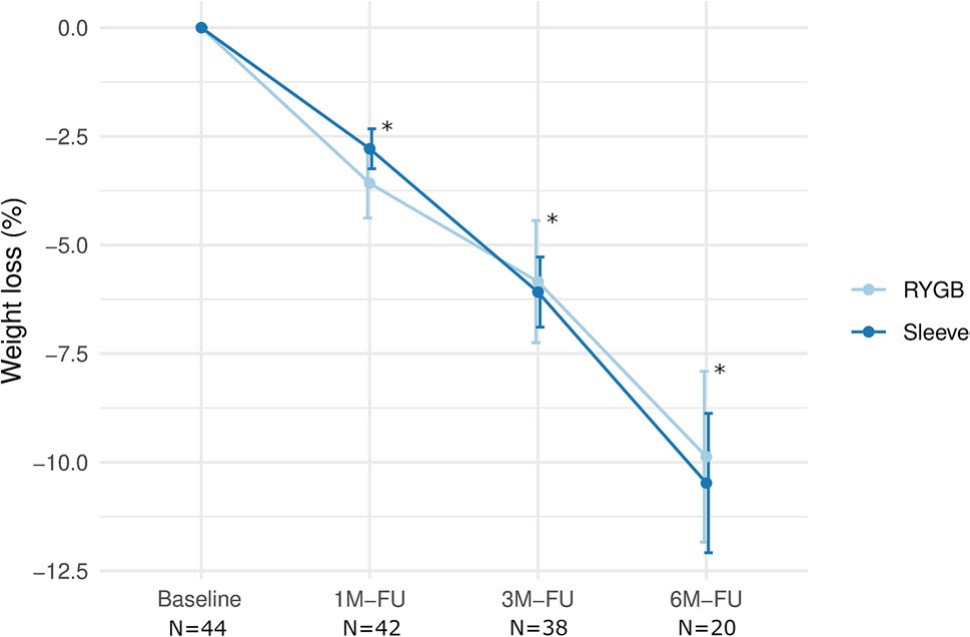
Weight loss over time following adjunct treatment with semaglutide once-weekly by type of surgery. SG, sleeve gastrectomy; RYGB, Roux-en-Y gastric bypass; FU, follow-up; N, number of individuals. Results are expressed as means and standard deviation. *Significantly different from baseline regardless of surgical group (*p* < 0.001)

With regard to type of surgery, there were no significant differences in semaglutide-induced weight loss after 3 months (*p* = 0.7) and 6 months (*p* = 0.8) between patients who had undergone SG and RYGB. BMI and change in BMI prior to initiation of semaglutide were not significantly different between the subgroups. In both subgroups, patients experienced a significant weight loss as early as 1 month post initiation of semaglutide (*p* < 0.001). Further reductions in body weight occurred at visit 2 (*p* < 0.001) and visit 3 (*p* < 0.001) compared to baseline (Fig. 1).

### Effects on Cardiovascular Surrogate Markers

Prior to semaglutide treatment, HbA1c was 5.3 ± 0.4% (mean ± SD); 8 patients suffered from prediabetes at baseline. Over the follow-up period, HbA1c remained stable. Also, there was no significant change in total cholesterol, HDL- and LDL-cholesterol, triglycerides, hs-CRP, and liver enzymes (Table 2).

**Table 2:** Anthropometric and biochemical characteristics at baseline and follow-up visits

	Baseline (*N* = 44)	3 M-FU (*N*** = 38)**	*p* value	6 M-FU (*N* = 20)	*p* value
Body weight [kg]	113.5 (25.2)	106.5 (24.5)	0.357	105.7 (25.1)	0.419
BMI [kg/m^2^]	38.3 (6.4)	36.0 (6.1)	0.147	36.2 (6.7)	0.380
Hb [g/dL]	13.4 (1.3)	13.6 (1.2)	0.703	13.4 (1.2)	0.999
Platelets [Mrd/L]	267.7 (71.3)	269.5 (60.8)	0.992	282.0 (111.9)	0.729
HbA1c [%]	5.3 (0.4)	5.2 (0.3)	0.310	5.2 (0.2)	0.446
Albumin [g/L]	38.8 (3.2)	39.3 (2.9)	0.999	38.5 (2.1)	0.999
Total cholesterol [mg/dL]	182.4 (35.6)	182.1 (37.0)	0.999	170.3 (28.6)	0.502
Triglycerides [mg/dL]	151.0 (68.8)	142.9 (64.2)	0.792	114.6 (38.7)	0.062
HDL-cholesterol [mg/dL]	55.3 (15.1)	51.7 (14.3)	0.471	50.4 (16.7)	0.394
LDL-cholesterol [mg/dL]	95.4 (30.9)	98.7 (33.8)	0.859	89.5 (27.9)	0.723
Total cholesterol/HDL-cholesterol ratio	3.4 (1.0)	3.8 (1.5)	0.346	3.9 (1.9)	0.328
AST [U/L]	21.7 (6.5)	24.0 (10.8)	0.555	24.3 (17.5)	0.600
ALT [U/L]	23.2 (10.1)	21.1 (12.2)	0.645	20.8 (13.8)	0.678
GGT [U/L]	20.6 (14.9)	18.7 (13.7)	0.796	16.8 (16.4)	0.549
CRP [mg/L]	4.1 (5.4)	4.0 (5.6)	0.998	5.8 (13.5)	0.640
hs-CRP [mg/dL]	0.3 (0.4)	0.3 (0.5)	0.999	0.2 (0.3)	0.603
Lipase [U/L]	42.5 (13.3)	43.5 (14.5)	0.928	44.4 (12.6)	0.834
Total weight loss [%]		− 6.0 (4.3)	< 0.001	− 10.3 (5.5)	< 0.001
Prediabetes	8 (18%)	2 (6%)	0.098	1 (5%)	0.179

Data are reported as mean (SD). *N*, number of individuals; *Hb*, hemoglobin; *HDL*, high-density lipoprotein; *LDL*, low-density lipoprotein; *AST*, aspartate aminotransferase; *ALT*, alanine aminotransferase; *GGT*, gamma-glutamyl transpeptidase; *CRP*, C-reactive protein; *hs-CRP*, high sensitive C-reactive protein

### Logistic Regression Analysis for Weight Loss of at Least 5% at 3 Months’ Follow-up

Triglycerides (OR = 0.99; *p* < 0.05), ALT (OR = 0.87; *p* = 0.05), and AST (OR = 0.89; *p* < 0.05) at baseline were negatively associated with weight loss of at least 5% at 3 months’ follow-up. Results of logistic regression analysis are shown in Table 3.

**Table 3:** Univariate logistic regression analysis to identify significant factors of successful weight loss defined as relative weight loss of ≥ 5% at 3 months’ follow-up compared to the baseline visit

Baseline visit data	OR	95% CI	*p* value
Age [year]	0.97	0.89–1.04	0.399
Sex [f]	4.44	0.95–25.09	0.067
Body weight [kg]	1.00	0.98–1.03	0.798
BMI [kg/m^2^]	1.10	0.99–1.26	0.110
Hb [g/dL]	0.69	0.37–1.15	0.180
Platelets [Mrd/L]	1.01	1.00–1.02	0.099
HbA1c [%]	6.01	0.85–69.77	0.102
Albumin [g/L]	0.77	0.55–1.01	0.081
Total Cholesterol [mg/dL]	1.00	0.98–1.02	0.766
Triglycerides [mg/dL]	0.99	0.97–1.00	0.016*
HDL-cholesterol [mg/dL]	1.01	0.97–1.06	0.503
LDL-cholesterol [mg/dL]	1.00	0.98–1.03	0.771
Total cholesterol/HDL-cholesterol ratio	0.72	0.35–1.42	0.353
AST [U/L]	0.89	0.78–0.99	0.047*
ALT [U/L]	0.87	0.78–0.95	0.005**
GGT [U/L]	0.99	0.94–1.04	0.627
CRP [mg/L]	1.06	0.92–1.31	0.498
hs-CRP [mg/dL]	1.75	0.35–18.65	0.552
Lipase [U/L]	0.97	0.92–1.02	0.200
Type of surgery [SG]	0.94	0.23–3.68	0.927
BMI before BS [kg/m^2^]	0.98	0.91–1.06	0.657
BMI nadir post BS [kg/m^2^]	1.07	0.97–1.22	0.206
Time from BS to weight loss nadir [months]	0.98	0.94–1.00	0.301
Time from BS to initiation of semaglutide treatment [months]	1.00	0.98–1.01	0.751
Total weight loss from BS to nadir [%]	1.04	0.98–1.10	0.248
Weight regain from nadir to initiation of semaglutide treatment [%]	1.01	0.96–1.06	0.747

Odds ratio (OR) with 95% confidence intervals (CI) and *p* values. *F*, female; *Hb*, hemoglobin; *HDL*, high-density lipoprotein; *LDL*, low-density lipoprotein; *AST*, aspartate aminotransferase; *ALT*, alanine aminotransferase; *GGT*, gamma-glutamyl transpeptidase; *CRP*, C-reactive protein; *hs-CRP*, high sensitive C-reactive protein; *SG*, sleeve gastrectomy; *BS*, bariatric metabolic surgery

## Conclusion

**Fig. 2:**
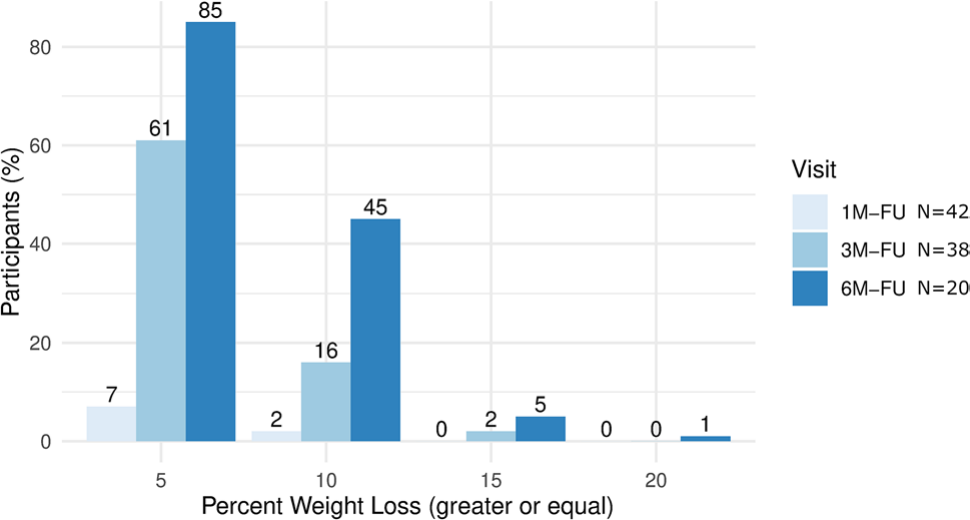
Percentages of patients who reached weight loss of at least 5%, 10%, 15%, and 20%, respectively, following adjunct treatment with semaglutide once-weekly for 1, 3, and 6 months. FU, follow-up; N, number of individuals

Here, we report on the effectiveness of adjunct medical treatment with semaglutide once-weekly in non-diabetic patients with WR or IWL after bariatric metabolic surgery. After 3 months (3.2, IQR 3.0–3.5, *n* = 38) of treatment with semaglutide, total weight loss was − 6.0 ± 4.3% (mean ± SD, *p* < 0.001) and after 6 months (5.8, IQR 5.8–6.4, *n* = 20), total weight loss added up to − 10.3 ± 5.5% (mean ± SD, *p* < 0.001). After 6 months of adjunct semaglutide treatment, 85% of patients reached > 5% weight loss, 45% of patients reached > 10% weight loss, and 5% of patients reached > 15% weight loss (Fig. 2). Side effects included gastrointestinal symptoms within 14 days of treatment initiation like nausea and a feeling of fullness, which was usually mild and did not lead to treatment discontinuation.

Analogous to the results of the semaglutide Phase III trial STEP-1 (the Semaglutide Treatment Effect in People with Obesity), post-bariatric patients of our cohort showed more than a 2% reduction in body weight within only the first 4 weeks of treatment initiation with semaglutide and continued to lose weight throughout the 6-month follow-up period [16]. Since semaglutide 2.4 mg once-weekly, which is a FDA/EMA-approved weight loss drug in the USA and Europe, was not yet approved for the treatment of obesity at the time the study was conducted, patients of our cohort received a maximum dose of 0.5 mg once-weekly.

There is limited data about the use of GLP-1 RA in post-bariatric WR or IWL, which were found to be more effective for treating post-bariatric weight regain than non-GLP-1 RA–based pharmacotherapies regardless of surgery type [21]. According to a retrospective review comparing amphetamine-derived phentermine with phentermine-topiramate, phentermine and phentermine-topiramate in addition to diet and exercise in post-RYGB and LAGB patients induced weight loss of 6.35 kg (12.8% excess weight loss) and 3.81 kg (12.9% EWL) within 90 days, respectively [14]. Zoss et al. reported weight loss of 8.0 ± 2.0 kg in patients with gastric banding treated with the lipase inhibitor orlistat 120 mg three times a day (TID) for 8 months [15]. A 12-month treatment with the GLP-1 RA exenatide twice a day (BID) resulted in more significant weight loss (− 14 kg) and diabetes resolution in a patient with gastric banding [22]. Also, a recent study in 117 patients without T2D suggests that post-bariatric surgery patients can lose a significant amount of weight (− 6.3 ± 7.7 kg, *p* < 0.05) within 4 months while taking liraglutide 3.0 mg regardless of the type of surgery they had [19]. Data from Pajecki et al. of 15 patients with weight regain after different surgical procedures point toward the same direction. Notably, all patients treated with liraglutide (max. dose 1.8 mg/day) reported improvement in satiety [23]. However, according to our data, the effectiveness of semaglutide seems to be superior to liraglutide 3.0 mg in post-bariatric WR or IWL. Differentiating between patients with WR and IWL, no significant differences in weight loss response were found. With respect to sex, female patients presented a more pronounced weight loss, which was statistically significant for the 3-month follow-up visit. Women seem to perform better in terms of EWL% following BS [24]. In contrast, a recent matched-pair cohort analysis of 707 men and women demonstrated that BS results in comparable short- and mid-term efficacy in men and women, and is associated with similar rate and severity of postoperative complications between sexes [25]. With respect to pharmacotherapy, especially GLP-1-based weight loss pharmacotherapy, the data are more consistent supporting the hypothesis of a gender‐dimorphic response with more pronounced weight reduction in females [26]. There is evidence that central GLP‐1 effects could be modulated by sex steroids; e.g., female rats are more sensitive than males to the anorexigenic effect of a centrally administered GLP‐1 receptor agonist. The anorexigenic effect of estrogen and modulation of GLP‐1 activity could involve the ventral tegmental area (VTA) and the nucleus accumbens (NAc) [26, 27]. Further studies are urgently needed to identify gender-related differences in efficacy and toxicity of GLP-1 RA in the pre- and post-bariatric population for a tailored approach to obesity management.

WR appears to differ by surgical procedure and tends to be higher in patients with SG [28]. Some reports have suggested that RYGB increases postprandial GLP-1 to a greater extent than SG [29]. Even though one could argue that enhancing the GLP-1 response could therefore be beneficial in patients who had SG, we did not observe any differences in the effectiveness of semaglutide once-weekly between SG and RYGB. This is in line with data from Wharton et al. who did not detect any differences in the effectiveness of liraglutide 3 mg 1 year after surgery [19]. In contrast, the observed response to adjuvant weight loss medication with phentermine, phentermine-topiramate extended release, lorcaserin, or naltrexone/bupropion was significantly better in gastric bypass and gastric banding patients compared with SG according to an observational study in 209 patients 1 year following BS. Furthermore, adjuvant pharmacotherapy was more effective in patients with higher BMI [30]. In our cohort, BMI categories prior to initiation of semaglutide and change in BMI were not significantly different between the surgical groups. Hence, we can only speculate that differences in initial BMI and change in BMI following pharmacotherapy would have driven greater weight loss in those patients with SG. According to available data in non-surgical patients, early treatment response (1–3 months) to the weight loss drug seems to be a good predictor of long-term weight maintenance [31–34]. Metabolic predictors of weight loss could not have been identified in patients treated with exenatide twice daily subcutaneously [35]. Predictors of response to pharmacotherapy in our cohort might be triglycerides, ALT, and AST at baseline, which were negatively associated with weight loss of at least 5% at 3 months’ follow-up. Even though there usually is remarkable variability in individual capacity for weight loss in response to pharmacological agents [36], these results might suggest that patients with poor metabolic long-term outcomes following BS are less likely to benefit from add-on pharmacotherapy.

Our study has a few limitations. Here we report a retrospective analysis of a small number of patients lacking a control group. At 6 months’ follow-up, only 20 patients have been included into the analysis. Differences in dose titration and time point of treatment initiation may have impacted the variability in weight loss.

To enhance outcomes for BS patients, close postoperative follow-up is required to address IWL or WR preferably early. Our study highlights a new and safe concept to pharmacologically treat IWL or WR along with lifestyle intervention following BS. Prospective randomized controlled trials are needed to evaluate larger cohorts of patients to determine if semaglutide once-weekly may close the gap between lifestyle intervention and revision surgery to treat weight recidivism or insufficient weight loss after BS.
